# An individual differences analysis of the neurocognitive architecture of the semantic system at rest

**DOI:** 10.1016/j.bandc.2016.07.003

**Published:** 2016-11

**Authors:** Giovanna Mollo, Theodoros Karapanagiotidis, Boris C. Bernhardt, Charlotte E. Murphy, Jonathan Smallwood, Elizabeth Jefferies

**Affiliations:** a Department of Psychology and York Neuroimaging Centre, University of York, Heslington, York, United Kingdom; b McConnell Brain Imaging Centre, Montreal Neurological Institute, McGill University, Montreal, QC, Canada

**Keywords:** Functional connectivity, Anterior temporal lobe, Inferior frontal gyrus, Visual word form area, Semantics, Verbal fluency

## Abstract

Efficient semantic cognition depends on accessing and selecting conceptual knowledge relevant to the current task or context. This study explored the neurocognitive architecture that supports this function by examining how individual variation in functional brain organisation predicts comprehension and semantic generation. Participants underwent resting state functional magnetic resonance imaging (fMRI) and, on separate days, performed written synonym judgement, and letter and category fluency tasks. We found that better synonym judgement for high frequency items was linked to greater functional coupling between posterior fusiform and anterior superior temporal cortex (aSTG), which might index orthographic-to-semantic access. However, stronger coupling between aSTG and ventromedial prefrontal cortex was associated with poor performance on the same trials, potentially reflecting greater difficulty in focussing retrieval on relevant features for high frequency items that appear in a greater range of contexts. Fluency performance was instead linked to variations in the functional coupling of the inferior frontal gyrus (IFG); anterior IFG was more coupled to regions of primary visual cortex for individuals who were good at category fluency, while poor letter fluency was predicted by stronger coupling between posterior IFG and retrosplenial cortex. These results show that individual differences in functional connectivity at rest predict semantic performance and are consistent with a component process account of semantic cognition in which representational information is shaped by control processes to fit the current requirements, in both comprehension and fluency tasks.

## Highlights

• Variations in semantic performance are reflected in resting-state networks.
• Inferior frontal connectivity predicts verbal fluency performance.
• Connectivity between visual and anterior temporal areas predicts synonym judgement.

## 1 Introduction

Semantic cognition has a central role in behaviour since it allows us to understand the meanings of words and objects around us and to use this conceptual knowledge to perform complex goal-orientated acts. Theories of semantic cognition emphasise that this capacity depends on multiple interacting components, supported by different neural processes (Jefferies, 2013, Jefferies and Lambon Ralph, 2006, Lambon Ralph, 2014). Although the extent to which visual, auditory and motor regions support semantic knowledge is still a matter of debate (Hauk and Tschentscher, 2013, Meteyard et al., 2012), a wealth of studies provide evidence that these brain regions contribute to our knowledge of what things look and sound like, and how we hold and use objects (Martin and Chao, 2001, Pulvermuller, 2001, Pulvermuller and Fadiga, 2010, Thompson-Schill, 2003). Anterior regions of the temporal lobe are thought to bring these different aspects of knowledge together to form amodal conceptual representations, allowing us to understand that items such as ‘kiwi’ and ‘pineapple’ are members of the same category even though they are different colours, sizes, shapes, have different textures, and are associated with different actions (Lambon Ralph et al., 2009, Lambon Ralph et al., 2010, Patterson et al., 2007, Rogers et al., 2004). Finally, left ventral and lateral prefrontal regions, as well as posterior middle and inferior temporal cortex, are important when conceptual information must be retrieved in the absence of strong contextual support, when there is strong competition from competing meanings, or when non-dominant aspects of meaning must be brought to the fore: for example, understanding that “kiwi” can refer to a bird as well as fruit (Badre et al., 2005, Jefferies, 2013, Noonan et al., 2013, Thompson-Schill et al., 1997, Wagner et al., 2001, Whitney et al., 2011).

Semantic cognition, therefore, reflects our ability to use conceptual information in a flexible way to serve different purposes. We retrieve semantic information to make sense of the environment around us, and also to generate thoughts and actions. Consequently, we need to be able to differentially engage different components of semantic cognition that support the current task demands (Badre et al., 2005, Jefferies and Lambon Ralph, 2006). First, in order to understand the significance of words and objects that we encounter in the external world, we need to be able to access relevant semantic representations from our sensory systems: for example, the comprehension of written words is thought to utilise mappings between visual responses in posterior fusiform cortex (encompassing the so-called ‘visual word form area’) and conceptual representations in anterior temporal cortex (Carreiras et al., 2014, Dehaene et al., 2010, Moore and Price, 1999). The nature of the stimulus can affect the efficiency of this visual-to-semantic transformation. For instance, highly imageable words, that rapidly arouse mental images associated with their meaning, enjoy a processing advantage compared to words that are less imageable. This advantage occurs because highly imageable words benefit from richer semantic associations (Plaut and Shallice, 1993, Wiemer-Hastings and Xu, 2005). Similarly, high frequency words that are often encountered benefit from a stronger mapping between orthography and meaning that is reflected in faster reading times (Balota et al., 2004, Chen et al., 2015). However, this type of semantic “access” may not be sufficient for good performance on tasks such as synonym judgement. This is because for any given concept, we have a multitude of knowledge and only a subset of this information is relevant for any given context. In order to correctly match words on the basis of their shared features (e.g., kiwi with tomato), semantic retrieval must be channelled to focus on relevant elements and away from strong functional associations (tomato goes with cheese sandwich). High frequency words are thought to require this type of control to a greater extent since they occur in multiple contexts and thus have a higher ‘contextual diversity’ (Almaghyuli et al., 2012, Hoffman et al., 2013, Hoffman et al., 2011).

There may be some differences in the neurocognitive components that are engaged when semantic information must be generated internally, as opposed to accessed from an external input (although both situations are thought to recruit conceptual representations in the anterior temporal lobes) (Adlam et al., 2010, Bozeat et al., 2000). In fluency tasks, conceptual information must be generated from a cue such as a letter or category name; here, the capacity to search for and select relevant knowledge is paramount. It is hypothesised that this process depends on the co-operation of the representational and control systems and draws heavily on left inferior frontal gyrus (Heim et al., 2008, Wagner et al., 2014). Moreover, the type of cue influences the extent to which control is required. Letter fluency, in which participants attempt to generate words starting with a particular letter, is particularly demanding of generation and selection mechanisms, while generating items from a category name such as “animals” requires less control, since a process of spreading activation between concepts will elicit high frequency and/or prototypical animals (Costafreda et al., 2006, Katzev et al., 2013). Recent work has shown that category fluency is more impaired in patients with degradation of conceptual representations following anterior temporal atrophy, while letter fluency is more vulnerable to poor semantic control (Rogers, Patterson, Jefferies, & Lambon Ralph, 2015). Moreover, category fluency appears to activate a broader range of sites implicated in internally-focussed memory retrieval, particularly retrosplenial cortex, while letter fluency has a clear prefrontal focus (Davies et al., 2004, Perani et al., 2003, Ryan et al., 2008, Shapira-Lichter et al., 2013).

Since comprehension and generation tasks require the components of semantic cognition to be brought together differently, we might anticipate that individual differences in these capacities should depend on different patterns of neural coupling that emerge at rest. This individual difference approach has been used successfully to understand the neural basis of various features of higher order cognition including meta-cognition, binocular rivalry, intelligence, reading comprehension and spontaneous thought (Baird et al., 2013, Baker et al., 2015, Gorgolewski et al., 2014, Smallwood and Andrews-Hanna, 2013, Smallwood et al., 2016, Xu et al., 2015). Few studies have attempted to link individual differences in semantic performance to the strength of resting state connectivity patterns. The most relevant study is by Wei et al. (2012), who found that stronger connectivity between posterior middle temporal gyrus and other parts of the semantic network, such as anterior temporal lobes and inferior frontal gyrus, predicted good performance on picture and sound naming and association judgements in a sample of 34 participants.

In the current study, we recorded resting state fMRI in a cohort of 48 participants who performed a series of tasks tapping different aspects of semantic performance on a subsequent day. This second experimental phase included a synonym judgement task to index the capacity to understand the meaning of an external stimulus (Jefferies, Patterson, Jones, & Lambon Ralph, 2009) and semantic and letter fluency tasks that required participants to internally generate representations. We explored how variation in participants’ performance on these tasks was related to resting state connectivity between regions previously implicated in written comprehension and fluency. This allows us to test the diagnostic value of resting state fMRI in the domain of individual differences in semantic cognition.

### 1.1 Regions of interest

Reflecting the component process account of semantic cognition above, we selected regions for our analysis that are implicated in (i) semantic representation (in the anterior temporal lobes), (ii) access to semantics from orthographic input (in left posterior fusiform), and (iii) lexical selection and semantic control (in inferior frontal gyrus). Previous fMRI studies of verbal semantic tasks have observed two distinct peaks in left anterior temporal lobe (ATL), in anterior superior temporal gyrus (aSTG) and in ventral ATL respectively (Binney et al., 2010, Hoffman et al., 2015, Schwartz et al., 2011, Visser et al., 2012, Visser and Lambon Ralph, 2011). Ventral ATL might provide a multimodal semantic hub anticipated by Patterson et al. (2007), since it responds across tasks and modalities (e.g., to pictures, environmental sounds, spoken and written words; Binney et al., 2010, Visser and Lambon Ralph, 2011; Visser, Embleton, Jefferies, Parker, & Lambon Ralph, 2010; Rice, Lambon Ralph, & Hoffman, 2015; Humphreys, Hoffman, Visser, Binney, & Lambon Ralph, 2015). Ventral ATL is functionally connected with semantic and default mode regions (Binney et al., 2010, Hoffman et al., 2015, Jackson et al., 2016, Pascual et al., 2015, Spitsyna et al., 2006). However, magnetic susceptibility artefacts produce signal loss and distortion in this region in standard EPI sequences, which mean it is consequently under-represented in the fMRI literature (compared with studies employing PET; Visser, Jefferies, & Lambon Ralph, 2010). In contrast, aSTG is less affected by magnetic susceptibility artefacts and often shows strong peaks in verbal comprehension tasks (Binney et al., 2010, Hoffman et al., 2015, Spitsyna et al., 2006), including studies employing the synonym judgements task used here (Binney et al., 2010, Hoffman et al., 2015). This region is functionally connected with auditory, somatosensory and other language-related regions (Bajada et al., in press, Binney et al., 2010, Jackson et al., 2016, Pascual et al., 2015).

In addition to these sites in ATL, we selected a region of left posterior fusiform cortex, often activated by orthographic stimuli and sometimes referred to as the “visual word form area” (Cohen et al., 2000, Rauschecker et al., 2012). This region has been consistently shown to be functionally and anatomically connected with language areas (Bouhali et al., 2014) and regions in the dorsal attention network (Vogel, Miezin, Petersen, & Schlaggar, 2012). We expected the connectivity profile of this region to explain differences in performance specifically in the synonyms task that relies on mapping the orthographic form of a stimulus onto the word meaning.

Finally, we selected sites in left inferior frontal gyrus (IFG), implicated in the selection and production of words. Studies have revealed functional specialisation within left IFG, with posterior regions engaged by lexical selection and phonological tasks, while anterior regions contribute to the controlled retrieval of semantic information (Devlin et al., 2003, Gough et al., 2005, Poldrack et al., 1999, Snyder et al., 2007, Wagner et al., 2001, Xiang et al., 2010). Consequently, we expected that the connectivity profile of seeds in posterior and anterior IFG might explain individual differences in letter and category fluency tasks respectively. Moreover, since synonym judgement requires semantic information to be retrieved in a controlled fashion, we expected that aIFG might also explain aspects of this task related to control demands.

### 1.2 Specific aims

In summary, our study was set out to examine the diagnostic value of measuring functional connectivity at rest in understanding individual differences in semantic cognition. We selected regions whose behaviour was expected to be important for making sense of written input in the synonyms task (posterior fusiform) and selecting and producing words in the fluency task (posterior IFG). We also selected two regions in the anterior temporal lobe thought to be critical for supporting semantic representations (in ventral ATL and aSTG), plus a region implicated in semantic control (anterior IFG), whose functional coupling could be important in different types of semantic tasks.

## 2 Materials and methods

### 2.1 Participants

This study was approved by the Ethics committee of the York Neuroimaging Centre and participants provided written informed consent prior to their participation. They took part to the study in exchange for course credit or monetary compensation. Participants were English native speakers, right handed, with normal or corrected-to-normal vision and no history of neurological or psychiatric disease.

The main study involved 48 participants (Group A; 14 men, age range 18–25 years). Five participants were excluded from the analysis due to technical problems affecting the behavioural tasks (N = 2), performance identified as outlier in the behavioural tasks (N = 1) or insufficient brain coverage (N = 2). The final sample of Group A included 43 participants (11 men, mean 20.3 ± 1.2 years).

Resting-state fMRI data from 20 participants in another experiment (Group B; 9 men, mean 23.8 ± 4.6 years) provided an independent repository with which to explore the networks underpinning the results observed for the group level regressions from Group A.

### 2.2 Experimental design and procedures

Members of Group A participated in three experimental sessions taking part in three separate days. They underwent a resting state functional and structural MRI scan during Session 1 and performed a series of computer-based tasks outside the scanner in Sessions 2 and 3, including synonym judgement and verbal fluency. Fluency and synonyms were both assessed in Session 2, with the fluency task performed first. Group B took part in a single session, starting with a resting state fMRI scan, followed by a task-based fMRI scan. The present study only used the resting state data from this sample.

### 2.3 Task stimulus materials and procedures

During *Verbal Fluency* (from Cambridge Semantic Battery; (Adlam et al., 2010, Bozeat et al., 2000), participants had 1 min to generate as many unique words as possible belonging to a semantic category (category fluency) or starting with a specific letter (letter fluency). Semantic fluency was assessed for eight categories split in two blocks (Block A: animals, fruits, birds, type of dogs; Block B: vehicles, tools, household objects, boats). Letter fluency was assessed for three letter cues (Block C: A, F, S). Block order was counterbalanced across participants and the order of cues within each block was randomized. Participants’ verbal responses were collected and the audio recordings were transcribed and scored off-line.

The *Synonyms Task* comprised 96 trials split into six conditions according to lexical frequency (high and low) and imageability (high, medium and low), details about this task can be found in Jefferies et al., 2009. All of the words in each trial fell into the same frequency and imageability condition. Each trial started with a fixation cross for 1 s, followed by a trial which remained on screen until the participant responded. A probe word was presented at the top of the screen (e.g., *STONE*) with the target and two unrelated distracters on the bottom row (e.g., *ROCK*, *WINTER*, *BOTTLE*). The words were written in black Arial font, size 18, on a white background. Participants were asked to select among the three choices the word closest in meaning to the probe. Responses were collected using the numeric keyboard.

For the purposes of the resting state functional connectivity analysis, participants’ performance in each task was evaluated by subtracting z-scored reaction times (RT) from z-scored accuracy. This *efficiency score* controls for speed accuracy trade-offs in a single measure. Positive efficiency scores indicate better performance, as these values follow the subtraction of negative z-scores for RT (indicating faster responses than average), from positive z-scores for accuracy (indicating more accurate responses than average).

### 2.4 MRI data acquisition

Brain imaging data were acquired at the York Neuroimaging Centre using a GE 3T HDX Excite MRI scanner and an eight-channel phased array head coil tuned to 127.4 MHz. The parameters for the functional and structural recordings were the same for Group A and B. The imaging session started with a 9 min eyes-open resting state functional scan using a gradient single-shot echo planar imaging (EPI) sequence with repetition time (TR) 3000 ms, echo time (TE) minimum full, 180 volumes, flip angle 90°, voxel size 3 × 3 × 3 mm^3^, matrix size 64 × 64, field of view (FOV) 192 × 192 mm^2^, slice thickness 3 mm and 60 slices with an interleaved (bottom up) acquisition order. The structural data were recorded using a sagittal isotropic 3D fast spoiled gradient-recalled echo (3D FSPGR) structural T1 weighted scan with the following parameters: TR 7.8 ms, TE minimum full, flip angle 20°, matrix size 256 × 256, 176 slices, voxel size 1.13 × 1.13 × 1 mm^3^, FOV 290 × 290 mm^2^. For each participant, a high-resolution T1-weighted in-plane anatomical picture was also acquired using a fluid attenuated inversion recovery (FLAIR) in order to facilitate the co-registration of the functional data onto the structural images.

## 3 Analysis

### 3.1 Resting state functional connectivity analysis

#### 3.1.1 Pre-processing

Functional and structural data were pre-processed and analysed using FMRIB’s Software Library (FSL version 4.1, www.fmrib.ox.ac.uk/fsl). Individual FLAIR and T1 weighted structural brain images were extracted using BET (Brain Extraction Tool) (Smith, 2002). Structural images were linearly registered to the MNI-152 template using FMRIB’s Linear Image Registration Tool (FLIRT) (Jenkinson & Smith, 2001). The resting state functional data were pre-processed and analysed using the FMRI Expert Analysis Tool (FEAT). The individual subject analysis involved: motion correction using MCFLIRT (Jenkinson, Bannister, Brady, & Smith, 2002); slice-timing correction using Fourier space time-series phase-shifting; spatial smoothing using a Gaussian kernel of FWHM 6 mm; grand-mean intensity normalisation of the entire 4D dataset by a single multiplicative factor; highpass temporal filtering (Gaussian-weighted least-squares straight line fitting, with sigma = 100 s); Gaussian lowpass temporal filtering, with sigma = 2.8 s.

#### 3.1.2 Seed based functional connectivity analysis

Functional connectivity was measured by looking at the temporal correlation between our regions of interest and the rest of the brain. There are different methods for correcting for physiological noise during resting state regression. Following from our prior studies (e.g. Davey et al., 2016, Gorgolewski et al., 2014, Smallwood et al., 2016), we did not use global signal regression but instead implemented component correction recommended by Murphy, Birn, Handwerker, Jones, and Bandettini (2009) which involves the extraction of the principle components in the white matter and the ventricles and controlling for these for the analysis of individual resting state scans.

The time series from 3 mm radius spheres were extracted and used as explanatory variables in connectivity analyses at the single subject level. In each analysis, we entered 11 nuisance regressors; the top five principal components extracted from white matter (WM) and cerebrospinal fluid (CSF) masks based on the CompCor method (Behzadi, Restom, Liau, & Liu, 2007) and six head motion parameters. WM and CSF masks were generated from each individual’s high resolution structural image (Zhang, Brady, & Smith, 2001).

Seed based functional connectivity analysis for Group A was conducted for seeds in the frontal and temporal lobes in the left hemisphere. First, we selected two coordinates within left anterior and posterior Inferior Frontal Gyrus (IFG), implicated in verbal fluency and semantic control. These regions are differentially implicated in semantic and letter fluency (Costafreda et al., 2006, Heim et al., 2009, Wagner et al., 2014), and in the controlled retrieval and selection of semantic representations (Badre et al., 2005, Noonan et al., 2013). The seed locations we used were taken from a meta-analysis of semantic control (Noonan et al., 2013): the posterior IFG site responded to control demands across both semantic and phonological tasks (pIFG; MNI x/y/z: −47/21/18), while the anterior IFG site responded to semantic control more than phonological control (aIFG; MNI x/y/z: −43/38/−10, both converted from Talairach using Bioimage suite (Papademetris et al., 2006). Secondly, we examined two spheres in the left anterior temporal lobe (ATL), taken from a previous fMRI study that examined functional activation for the same synonym judgement task used in our investigation (Binney et al., 2010). This study revealed strong engagement of anterior Superior Temporal Gyrus (aSTG; MNI x/y/z: −57/6/−18), commonly activated by verbal semantic tasks in the wider literature (Hoffman et al., 2015, Spitsyna et al., 2006, Visser and Lambon Ralph, 2011), and some activation in ventral ATL, where activation is less commonly observed across studies (Visser, Jefferies et al., 2010). Binney et al. (2010) used a novel fMRI sequence designed to overcome magnetic susceptibility artefacts in ventral anterior temporal regions. We did not observe task effects relating to the ventral ATL seed, perhaps because we did not use methods designed to minimise signal loss and distortion at this site: thus ventral ATL is not discussed further below. Finally, we examined a region in the posterior fusiform cortex known as the Visual Word Form Area (VWFA; MNI x/y/z: −43/−57/−23; (Rauschecker et al., 2012). This region has been consistently shown to be involved in the identification of written words (Cohen et al., 2000).

For Group A, the statistical group-level analyses were carried out using FMRIB’s Local Analysis of Mixed Effects (FLAME1). The group-level analyses included a series of multiple regressions using the connectivity maps for each seed region as the dependent variable and the participants’ performance as the independent variable. Separate regression models were run for each task and for each seed.

For the Synonyms Task, we employed separate models examining differences in performance relative to frequency (conditions: high and low frequency items) and imageability items (conditions: high and low imageability items - the medium imageability items were disregarded). For Fluency, we included Category and Letter Fluency conditions in the same model. The contrasts explored the correlation between the functional connectivity maps of each seed and (a) good or bad performance at each condition, (b) good or bad performance at the task, plus (c) differential effects of the conditions (HF vs. LF and HI vs. LI words in the synonym task; letter vs. category fluency).

The nature and interpretation of correlation in resting state analysis is a matter of a debate that is focused on a lack of clarity regarding what constitutes a correlation of zero (see Murphy et al., 2009). Our results describe the beta weights that are produced through the process of multiple regression and reflect a significant positive or negative difference relative to the z-scored distribution of correlations in the whole brain. In other words our analysis allows the identification of regions that show relatively greater or relatively weaker correlations with the seed region. We therefore use the terms ‘relative strong’ and ‘relative weak’ correlated to describe regions whose correlation with the seed region is higher or lower than the average.

All analyses were corrected for multiple comparisons at a cluster-wise family-wise p < 0.05, using a z-statistic threshold of 2.3 to define contiguous clusters. In the multiple regressions analysis, we also controlled for the number of seed regions, as well as the two-tailed nature of our hypotheses, adopting a highly conservative alpha value of 0.00625.^1^ As this is likely to generate Type II errors, we also report statistically significant effects at the standard threshold of 0.05, as those results can help the interpretation of the effects observed at the more conservative threshold. Furthermore the unthresholded maps are made publicly available through Neurovault here: http://neurovault.org/collections/1424/.

To examine the functional architecture associated with the spatial maps that predicted behavioural performance, a second seed-based functional connectivity analysis was performed using data from Group B. Here, we seeded the spatial maps that correlated with behavioural performance from the original four seeds to recover their broader resting-state networks.

These statistical models include multiple predictors as explanatory variables and so any statistical results that emerge from these models are independent of the other explanatory variables. We formalised contrasts that captured these statistically independent results, as well as explicit contrasts that differentiate between the explanatory variables.

## 4 Results

### 4.1 Behavioural data

In the Synonyms Task, responses in high frequency trials were more accurate (t(42) = 12.73, p < 0.001) and faster (t(42) = −16.33, p < 0.001) than low frequency trials. Similarly, trials composed of high imageability words were more accurate (t(42) = 7.70, p < 0.001) and faster (t(42) = −7.45, p < 0.001) than low imageability trials.

In the Fluency Tasks, the number of correct words generated per minute was equivalent for Category and Letter Fluency (t(42) = 0.43, p = 0.67). There were more errors in Category than Letter fluency (t(42) = −5.23, p < 0.001). Descriptive statistics are shown in Table 1 while the correlations between the behavioural measures are shown in Table 2.

### 4.2 Neuroimaging results

**Fig. 1:**
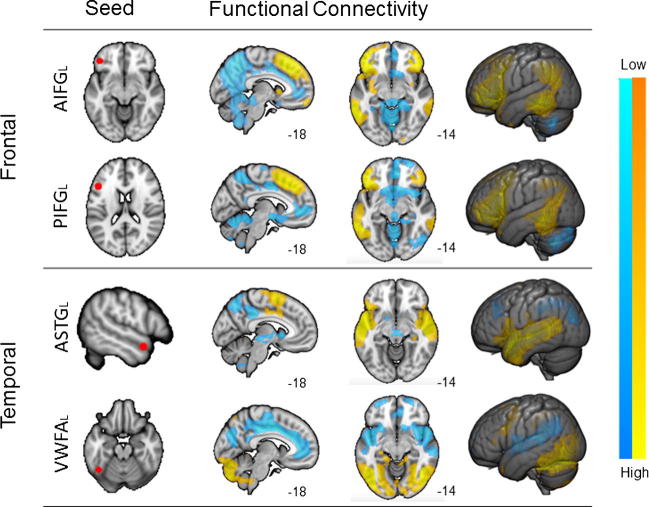
Seed based connectivity maps. This figure shows the results of a seed-based connectivity analysis from each of the seed regions. The location of the seeds is shown in the left most image in each row. Spatial maps were thresholded at Z < 2.3 and corrected at p < 0.05 FWE. The different colour schemes describe the strength of correlations with the seed regions.

In the resting state fMRI analysis, we calculated spatial maps corresponding to relatively strong correlation of the time series, and relatively weak correlation, for each seed region, presented in Fig. 1. Both aIFG and pIFG exhibited extensive bilateral connections to dorsal medial and lateral prefrontal cortex, as well as lateral regions of the posterior temporal cortex extending on the left hemisphere into the angular gyrus and lateral occipital cortex. This pattern of connectivity partially overlaps with the ‘fronto-parietal control network’ (Spreng, 2012, Yeo et al., 2011). Differences in the functional specialisation between these two regions are confirmed by the relatively strong connectivity of aIFG with bilateral insula and left anterior temporal lobe – a core region in the semantic system - and the stronger connectivity of the posterior seed with the left superior temporal sulcus involved in phonological processing (Xiang et al., 2010). In addition, both regions showed low correlation with the cingulate cortex and precuneus, this pattern was observed bilaterally for the anterior seed and predominantly on the right hemisphere for the posterior seed. The VWFA seed was strongly correlated with occipital regions and posterior and ventral temporal cortex, bilaterally. This region exhibited a pattern of connectivity usually identified as visual network and dorsal-attention network (Yeo et al., 2011). It also showed relatively weak correlation with medial temporal lobe, angular gyrus and cingulate cortex extending into ventral medial prefrontal cortex, bilaterally. The aSTG seed was strongly coupled to the temporal lobes and to regions of motor cortex, including supplementary motor cortex. This pattern is consistent with the connectivity profile of the anterior portion of the superior temporal cortex reported in previous studies (Hurley et al., 2015, Jackson et al., 2016, Pascual et al., 2015). This seed also showed relatively weak correlation with the ventral striatum, middle frontal gyrus, regions in the dorsal precuneus and angular gyrus, bilaterally. Table 3 provides a complete description of the regions passing cluster correction for all seed regions.

### 4.3 Relationship to behaviour

The next step in our analysis examined the relationship between the functional connectivity measures for each participant and their performance on synonym judgement and fluency. We implemented a series of multiple regressions using FLAME with the spatial maps generated from each seed as the dependent variable and the efficiency with which the participant performed each task as the independent variable. In order to determine the functional architecture associated with the cluster maps identified with the previous analysis, we subsequently seeded these cluster maps in an independent dataset (Group B).

#### 4.3.1 Synonyms task

**Fig. 2:**
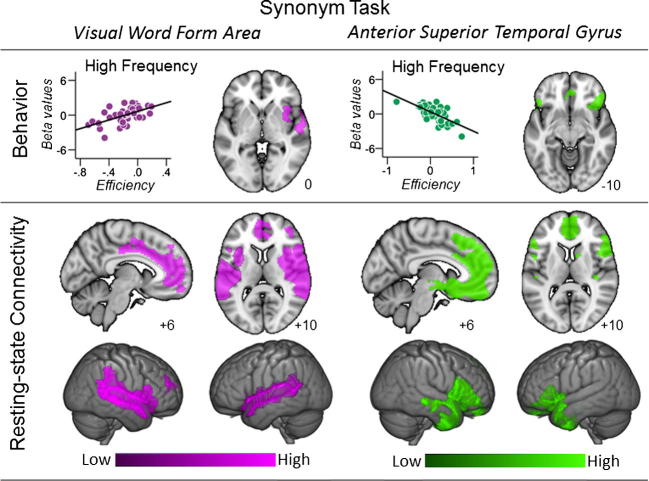
Synonyms task performance. This figure shows the results of group level regressions predicting performance on high and low frequency trials in the synonyms task from the connectivity maps generated from two of the seed regions in this experiment: the visual word form area (VWFA) and the anterior superior temporal gyrus (aSTG). This analysis shows that effective synonym performance was associated with (i) stronger coupling between the VWFA and the anterior temporal lobe (represented in violet) and reduced coupling between the aSTG seed and ventral regions of the medial prefrontal cortex (represented in green). In the upper panel, the scatter plots show the relationship between synonym efficiency and the connectivity with the relevant region and the axial slice shows the clusters spatial location (Group A). Each point on the scatter plots is an individual participant. The lower panel shows the results of seeding the clusters generated in the group level regressions (Group B). Spatial maps were thresholded at Z < 2.3 and corrected at p < 0.05 FWE, accounting for the number of seed regions (n = 4) and the number of voxels in the cortex. In this figure different colours represent different seed regions.

**Fig. 3:**
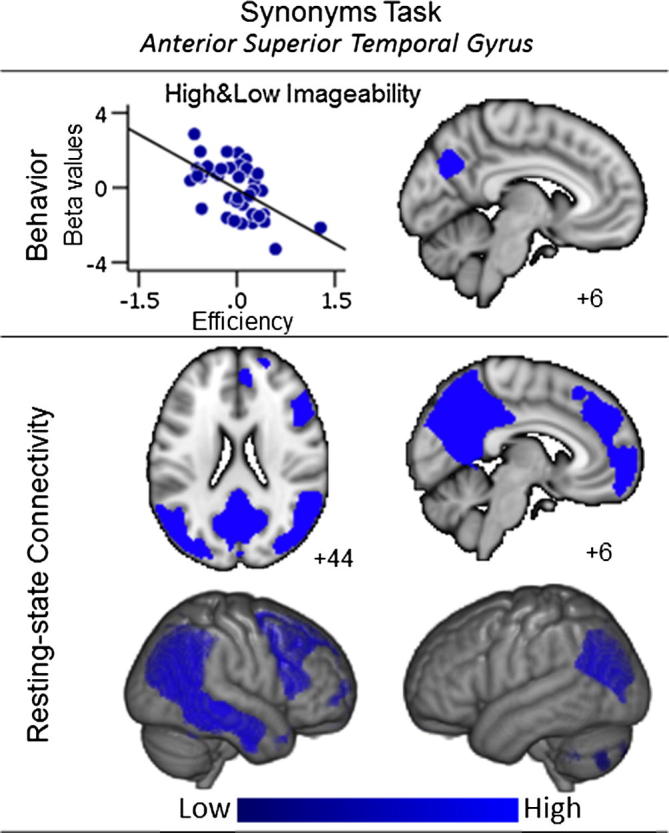
Synonyms task performance. This figure shows the results of group level regressions predicting performance on the high and low imageability trials in the synonyms task from the connectivity maps generated from the seed regions in this the anterior superior temporal gyrus (aSTG). This analysis shows that overall effective synonym performance was associated with worse coupling between the aSTG seed and a regions of the posterior cingulate cortex (represented in blue). In the upper panel, the scatter plots show the relationship between synonym efficiency and the connectivity with the relevant region and the axial slice shows the clusters spatial location (Group A). Each point on the scatter plots is an individual participant. The lower panel shows the results of seeding the clusters generated in the group level regressions (Group B). Spatial maps were thresholded at Z < 2.3 and corrected at p < 0.05 FWE, accounting for the number of seed regions (n = 4) and the number of voxels in the cortex. In this figure different colours represent different seed regions.

We found a significant relationship between synonym performance and the connectivity of the two temporal lobe regions: VWFA and aSTG. These are presented in Fig. 2. Table 4 presents the magnitude and size of the clusters that were significant in these analyses. For the VWFA, we observed a region of right aSTG and anterior insula that was more coupled to the seed region for people who performed the high frequency trials with greater efficiency. This result could reflect more efficient semantic access from orthographic/visual processes to semantic representations in ATL. Seeding this region in an independent data set (Group B) revealed that it was functionally coupled to anterior and mid-cingulate cortex as well as bilaterally to the temporal lobe. In addition, poor performance on the high frequency trials of the synonym task was associated with stronger coupling between the aSTG seed and a region of ventral prefrontal cortex (vPFC, see Fig. 3 and Table 4). This same cluster also showed stronger coupling with aSTG for participants who showed relatively poor performance for high frequency vs. low frequency trials indicating that the pattern was a differential effect associated with performance specifically on high frequency items (Table 4). Subsequent seeding of this region in the data from Group B demonstrated that it was functionally coupled to the medial prefrontal cortex, ventral regions of the lateral prefrontal cortex and limbic regions including the ventral anterior temporal lobe which may promote a pattern of off-task semantic retrieval which could be especially disruptive for HF trials with higher contextual diversity and control demands.

Finally, stronger coupling between aSTG and precuneus was associated with poor synonym performance, on average, for the trials in the imageability analysis. The connectivity maps associated with this cluster, seeded in the data from Group B, included ventromedial and ventrolateral prefrontal regions and bilateral angular gyrus, a pattern that reflects the so-called default mode network (DMN) (Buckner et al., 2008, Raichle et al., 2001). This is presented in Fig. 3. This pattern of coupling suggests that connectivity between the aSTG and the posterior core of the DMN was associated with inefficient performance on the synonyms task in general.

#### 4.3.2 Fluency task

**Fig. 4:**
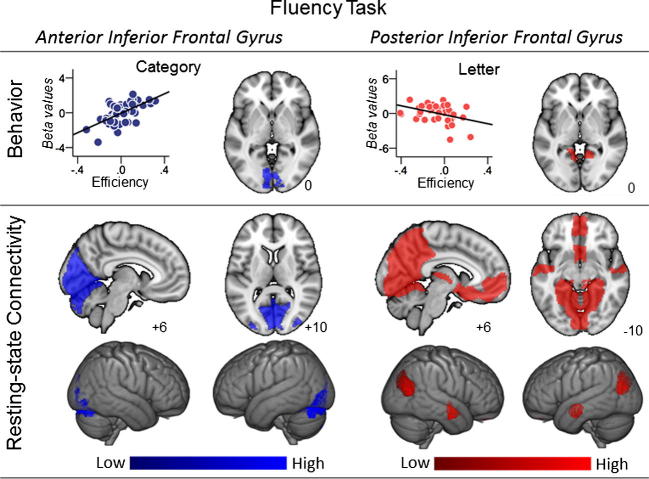
Fluency task performance. This figure shows the results of group level regressions predicting performance on letter and category trials in the fluency task from the connectivity maps generated from two of the seed regions in this experiment: the anterior Inferior Frontal Gyrus (aIFG) and posterior Inferior Frontal Gyrus (pIFG). This analysis shows that effective category fluency performance was associated with stronger coupling between the aIFG and medial regions of occipital cortex (represented in blue). By contrast, effective letter fluency was associated with reduced coupling between the pIFG seed and regions of the retrosplenial cortex (RSC) (represented in blue). It is important to note that the pattern of pIFG coupling pattern was sensitive to the nature of the cue since participants who performed well on the category fluency task showed higher coupling to this region. In the upper panel, the scatter plots show the relationship between synonym efficiency and the connectivity with the relevant region and the axial slice shows the clusters spatial location (Group A). Each point on the scatter plots is an individual participant. The lower panel shows the results of seeding the clusters generated in the group level regressions (Group B). Spatial maps were thresholded at Z < 2.3 and corrected at p < 0.05 FWE, accounting for the number of seed regions (n = 4) and the number of voxels in the cortex. In this figure different colours represent different seed regions.

Fluency performance was associated with greater connectivity from the prefrontal cortex seeds (see Fig. 4, Table 4). Superior performance on Category Fluency was associated with greater connectivity between the aIFG seed region and the medial occipital cortex. Seeding these regions in the data from Group B illustrated that this region was functionally coupled to primary visual areas in both hemispheres. Finally, greater efficiency on Category Fluency was also associated with stronger connectivity between aSTG and a cluster in the cerebellum, extending into ventral inferior temporal cortex bilaterally. These latter results are difficult to interpret because the cluster map crosses anatomical boundaries that are not directly linked (e.g. there are no direct links between primary visual cortex and the cerebellum, see also Smallwood et al., 2013 for a similar issue). For this reason, we won’t include them in the discussion but we made the unthresholded maps of these results publicly available on Neurovault (Table 5).

Poor performance on Letter Fluency was associated with greater connectivity between pIFG and the retrosplenial cortex (RSC). This cluster overlapped with a region that showed an effect of category > letter fluency that passed correction for multiple comparisons at family-wise error level of p < 0.05. Although this did not pass the alpha value that controls for the number of seed regions, this pattern allows us to reject the hypothesis that this increased connectivity was associated with problems in fluency per se – instead, the effect was a differential effect that was specific to poor Letter Fluency. Thus, stronger connectivity between IFG and RSC was associated with difficulty in efficiently generating words that started with a specific letter as opposed to items that were conceptually linked. Seeding of this cluster in the data from Group B demonstrated strong coupling between RSC and ventromedial cingulate/prefrontal cortex, as well as with anterior temporal lobes.

## 5 Discussion

The current study set out to investigate how variations in performance in tasks that emphasise different aspects of semantic cognition are reflected in the functional connectivity of the brain at rest. We found that connectivity of the left IFG was predictive of performance in fluency tasks, consistent with observations from functional neuroimaging and lesion studies showing that this region is activated in the generation of semantic information. We also found that synonym judgement performance was related to the connectivity of both the putative VWFA and aSTG, regions that are activated when participants perform similar tasks. Together these data indicate that individual differences in semantic performance can be related to the behaviour at rest of specific cortical regions implicated in semantic processing.

More generally, our results are consistent with the hypothesis that semantic cognition emerges through the flexible interaction of distributed and functionally independent components, including areas implicated in conceptual representation, access to semantics from vision and the capacity to generate and select information (Badre et al., 2005, Jefferies, 2013, Jefferies and Lambon Ralph, 2006, Noonan et al., 2013). Effective synonym judgement for high frequency words was linked to strong connectivity between the putative VWFA and regions of the ATL: this pattern might reflect greater coupling between temporo-occipital regions supporting visual/orthographic processing and anterior temporal regions representing the meanings of words. This effect was not apparent for fluency tasks that rely on the generation of information from memory rather than the translation of orthographic input. Instead, the ability to generate exemplars of a category was associated with stronger coupling between aIFG and the occipital cortex, a finding that is broadly consistent with accounts of semantic cognition that emphasise the contribution of visual and other sensory/motor regions to conceptual processing (Martin and Chao, 2001, Patterson et al., 2007, Pulvermuller, 2001). Specifically, in category fluency tasks, participants are asked to generate objects within a category that tend to have some overlap of their visual features – for example, animals all have legs, eyes, ears etc. Visual imagery or retrieval drawing on these primed features could therefore allow category exemplars to be generated more effectively.

We also found that effective generation in response to a letter cue, but not a category cue, was linked to reduced connectivity between pIFG and RSC. Recent functional evidence has shown that the RSC shows an increased response when participants generate information from categorical cues (Shapira-Lichter et al., 2013), perhaps because generating items in a spatial context facilitates the retrieval of more category members that are also found in the same context (e.g., thinking of a snake in the zoo helps the retrieval of more zoo animals). This interpretation draws on findings showing a response in RSC in situations in which context supports memory retrieval (Aminoff et al., 2013, Kveraga et al., 2011) and more generally through the role of this system in scene construction (Hassabis & Maguire, 2007). Letter fluency would not benefit from the application of context in the same way since items that start with the same initial letter are not typically found in the same context – indeed the generation of strong contextual or schematic information in this task could hinder performance. For example, thinking of snake in the zoo when generating items starting with *S* is likely to elicit competition from concepts related to snake that do not start with the appropriate letter.

Our results build on prior studies that have examined resting state networks linked to semantic processing (Hurley et al., 2015, Jackson et al., 2016, Pascual et al., 2015) by demonstrating differences in the functional coupling between components of the semantic network at rest can be related to differences in performance on a range of semantic tasks. This is consistent with the proposal that aspects of semantic cognition emerge through the flexible coupling of nodes within large-scale distributed networks (e.g. Jefferies, 2013). We found that poor performance across tasks (e.g., less efficient synonym judgement and poor letter fluency) was commonly linked to stronger engagement of default mode and limbic regions. Psychologically, letter fluency and synonym performance for high frequency words share a reliance on executive processes (Almaghyuli et al., 2012, Hoffman et al., 2011, Hoffman et al., 2013, Jefferies and Lambon Ralph, 2006, Martin et al., 1994, Rogers et al., 2015), so it is possible that this commonality may reflect the role that control processes play in semantic cognition. For example, some participants may have had more difficulty deploying task-appropriate strategies in the face of strong but irrelevant semantic links: for letter fluency, they may have engaged a search based on global associations, while for synonym judgement, they may have retrieved associations rather than concepts with shared features. Alternatively, some participants may have had difficulty constraining their attention to the task in hand, a state that is known to impact negatively on task performance (for a review see Smallwood & Schooler, 2015). This latter hypothesis is supported by the observation that the DMN (Buckner et al., 2008, Raichle et al., 2001) has an antagonistic relationship to executive regions (Fox & Raichle, 2007) and can derail task performance when activity occurs under inappropriate conditions (Smallwood et al., 2013, Weissman et al., 2006). There was a link between poor performance and stronger connectivity between language/semantic and default mode regions in several independent models (e.g., for letter fluency from pIFG, and high frequency words from aSTG): when the regions associated with poorer performance in these analyses were seeded in an independent data set, they showed common areas of functional connectivity in default mode and limbic cortex, most clearly in ventromedial PFC. Nevertheless, these findings do not contradict the view that, under some circumstances, greater engagement of regions within the DMN (e.g., regions in ATL that fall within this network) may show a positive relationship with semantic performance. It may be the specific nature of network-network coupling combined with the specific task demands that determine the consequence for behaviour (see also Smallwood et al., 2013).

In conclusion, these data demonstrate that performance on semantic tasks can be understood by investigating the functional architecture of the brain at rest. We found that certain features of semantic task performance are linked to patterns of stronger functional coupling, such as the increased temporal correlation between posterior fusiform (VWFA) and ATL which predicted better performance on synonym judgement trials employing high frequency words. Other aspects of semantic performance were linked to decreased coupling between regions, such as the reduced connectivity between the posterior inferior frontal gyrus and the retrosplenial cortex that was linked to worse letter fluency. These data support a component process account of semantic cognition in which semantic retrieval emerges through the flexible interaction of different nodes within a distributed semantic network. One important aim for future studies will be identifying the extent to which there are patterns of resting state activity that are common to particular semantic tasks and others that discriminate between them. It would also be useful to examine how these putative semantic networks at rest are related to the spatial extent of the same networks as localised by online semantic task performance, allowing similarities and differences in the behaviour of semantic cognition networks to be characterised at rest and during tasks (for an example of this see Davey et al., 2016, Krieger-Redwood et al., submitted for publication). Our method may also aid the assessment of semantic cognition in populations such as children or patients, when measuring task performance can be problematic.

We conclude with the observation that since prior studies have identified a relationship between functional organisation at rest and the type of cognition that is experienced during the resting state (Gorgolewski et al., 2014, Smallwood et al., 2016, Tusche et al., 2014), some of the relationships that our study identified may reflect the expression of spontaneous thought when participants are not actively engaged with an externally-presented task. It seems plausible that particular types of spontaneous thought may recruit aspects of semantic cognition for their expression and elements of the neural coupling that we have identified at rest indicate these relationships. A future comparison of how connectivity patterns at rest relate to ongoing stimulus independent thoughts, and semantic task performance, could reveal the role that the semantic system plays in naturally occurring forms of thinking.

## Figures and Tables

**Table 1:** Behavioural results. Reaction times in the synonyms task are to correct trials only. Examples of synonyms pairs for each condition are reported in parenthesis (the probe is in italics). Accuracy scores for fluency show the percentage of responses that were appropriate to the category (with errors including both out of category responses and repetitions).

	% Accuracy (SD)	Words per minute (SD)
*Fluency task*		
Category fluency	0.91 (0.06)	14.8 (2.7)
Letter fluency	0.97 (0.03)	14.6 (3.2)

	% Accuracy (SD)	Reaction time in ms (SD)

*Synonyms task*		
High frequency (*Rock* – STONE)	0.98 (0.02)	1659 (315)
Low frequency (*Attribute* – TRAIT)	0.85 (0.07)	2589 (532)
High imageability (*Sun* –MOON)	0.94 (0.05)	1872 (302)
Low imageability (*Effect* – CONSEQUENCE)	0.85 (0.07)	2511 (677)

**Table 2:** Correlations between behavioural measures. Correlations between task performance computed using the efficiency scores (^*^ = 0.05; ^**^ = 0.01).

	Fluency task		Synonyms task			
			Frequency		Imageability	
	Category	Letter	High	Low	High	Low
Category fluency	1	0.354^∗^	0.154	0.308^∗^	0.199	0.328^∗^
Letter fluency		1	0.388	0.368^∗^	0.373^∗^	0.298
High frequency			1	0.471^∗^	0.598^∗∗^	0.483^∗∗^
Low frequency				1	0.604^∗∗^	0.876^∗∗^
High imageability					1	0.339^∗^
Low imageability						1

**Table 3:** List of clusters showing strong or weak functional connectivity at rest with each seed for Group A. Anatomical labels were provided by the Harvard-Oxford Atlas implemented in FSL view.

Anterior inferior frontal gyrus				Strong connectivity			
Cluster	Brain area	Voxels	P	Z-Max	x	y	z
1	L Frontal Pole	11852	>0.001	12.6	−44	38	−10
2	L MTG	5205	>0.001	8.13	−56	−46	−8
3	R Frontal Pole	4398	>0.001	7.71	40	40	−12
4	R Cerebellum	2333	>0.001	7.3	14	−82	−32
5	R MTG	719	0.020	4.66	68	−34	−8

				Weak connectivity			
1	R Precuneus	25006	>0.001	7.21	16	-62	24
2	R MFG	623	0.038	6.67	26	32	36

Posterior inferior frontal gyrus				Strong connectivity			

1	L IFG	9029	>0.001	12.4	−46	20	20
2	L MTG	6146	>0.001	7.12	−58	−48	−6
3	R IFG	2163	>0.001	7.05	54	26	16
4	R Cerebellum	1933	>0.001	7.07	16	−78	−34
5	R MTG	647	0.041	4.44	66	−48	−4

				Weak connectivity			
1	R Cingulate Gyrus	17807	>0.001	6.94	6	44	0
2	R Caudate	3187	>0.001	5.45	14	24	0
3	R Cerebellum	790	0.017	4	52	-58	-34

Anterior superior temporal gyrus				Strong connectivity			

1	L Temporal Pole	8573	>0.001	12	−56	8	−16
2	R STG	6271	>0.001	8.57	48	−18	−10
3	L SMA	1376	>0.001	5.89	−4	0	62

				Weak connectivity			
1	L LOC	2207	>0.001	6.51	−36	−78	28
2	R MFG	1527	>0.001	5.07	−24	10	48
3	L Cingulate Gyrus	1503	>0.001	4.74	−8	-34	34
4	R Thalamus	1230	>0.001	5	2	−22	−6
5	R SFG	1163	>0.001	4.61	24	12	48
6	R Cerebellum	1147	>0.001	5	42	−68	−48
7	R LOC	1080	>0.001	5.23	42	−74	24

Visual word form area				Strong connectivity			

1	L Fusiform Gyrus	27130	>0.001	13	−44	−58	−24
2	L Precentral Gyrus	1617	>0.001	6.32	−46	4	26
3	R Precentral Gyrus	652	0.029	5.75	48	6	28

				Weak connectivity			
1	R Cingulate Gyrus	10784	>0.001	6.31	6	−22	36
2	R Supramarginal Gyrus	7375	>0.001	6.03	50	−36	22
3	L Planum Temporale	5539	>0.001	6.87	−44	−30	8
4	R Frontal Pole	1356	>0.001	4.59	24	44	28
5	L Frontal Pole	805	0.011	4.37	−32	48	−16

**Table 4:** List of clusters showing a significant association between a behavioural performance and functional connectivity at rest for each seed for Group A. This table reports all effects significant at the standard threshold of 0.05. Asterisks (^∗^) indicate the clusters surviving the adjusted alpha value of 0.005 which were seeded in Group B. Note that − (*minus*) indicates poor performance, + (*plus*) indicates good performance, ‘&’ indicates global task performance. Anatomical labels were provided by the Harvard-Oxford Atlas implemented in FSL view.

	Seed	Contrast	Brain area	Voxel	p	Z	x	y	z
Synonyms frequency	aSTG	HF – ^∗^	R Frontal Pole	2408	>0.001	5.2	40	20	−18
		HF –	R Frontal Pole	658	0.024	4.2	12	50	36
		HF&LF −	R Lateral Occipital	605	0.036	4.9	32	−78	30
		LF > HF ^∗^	L Frontal Orbital Cortex	1857	>0.001	4.4	−28	20	−22
	VWFA	HF + ^∗^	R STG	948	0.004	4.6	56	−10	−4
		HF > LF	RSTG	580	0.048	3.5	54	−10	−6

SynonymsI mageability	aSTG	HI − ^∗^	L Precuneus	884	0.006	3.7	−10	−72	28
		HI&LI − ^∗^	L Precuneus	940	0.004	4.1	−6	−68	36
		HI&LI –	R Lateral Occipital Cortex	673	0.023	4.5	34	−78	30

Letter and category fluency	aIFG	Cat + ^∗^	R Fusiform Gyrus	1151	0.001	4.1	22	−86	−14
		Cat > Let	L Post central gyrus	643	0.033	3.8	−6	−44	66
	pIFG	Cat −	R MTG	655	0.039	3.5	56	−60	8
		Let − ^∗^	R Precuneus	977	0.006	3.7	22	−56	14
		Cat > Let	L Lingual Gyrus	707	0.028	3.5	−16	−42	−6
	aSTG	Cat +	L Cerebellum	876	0.006	4.5	−10	−38	−32
		Cat +	R Fusiform Gyrus	756	0.013	4.2	28	−38	−28

**Table 5:** List of clusters showing strong functional connectivity at rest seeding the clusters generated in the group level regressions for Group A in an independent dataset (Group B). Anatomical labels were provided by the Harvard-Oxford Atlas implemented in FSL view.

Cluster	Brain area	Voxels	P	Z-Max	X	y	Z
*Cluster mask aIFG - category fluency*							
	L Lingual Gyrus	14556	>0.001	8.17	−6	−86	−14

*Cluster mask pIFG - letter fluency*							
2	R Precuneus	26136	>0.001	8.58	2	−54	12
1	R Angular Gyrus	1408	>0.001	6.04	52	−60	14

*Cluster mask aSTG - high frequency*							
1	L Frontal Orbital Cortex	26121	>0.001	7.5	−26	22	−22

*Cluster mask aSTG - high imageability*							
3	R Angular Gyrus	20038	>0.001	8.65	58	−58	22
2	R MFG	5806	>0.001	6.59	26	26	40
1	L Cerebellum	1230	>0.001	5.68	−42	−50	−42

*Cluster mask VWFA - high frequency*							
2	R Temporal Pole	18087	>0.001	7.35	48	12	−8
1	L Planum Polare	9409	>0.001	6.93	−48	4	−8

## Footnotes

1 Given difficulties in obtaining a reliable signal in the ATL we did not include this region in this calculation.
